# Enzymatic degradation of pea fibers changes pea protein concentrate functionality

**DOI:** 10.1016/j.crfs.2024.100744

**Published:** 2024-05-04

**Authors:** Joël I. Zink, Olivia Zehnder-Wyss, Dylan Dällenbach, Laura Nyström, Erich J. Windhab

**Affiliations:** a Laboratory of Food Process Engineering, Department of Health Science and Technology, ETH Zurich, Schmelzbergstrasse 9, Zurich, 8092, Switzerland; b Laboratory of Food Biochemistry, Department of Health Science and Technology, ETH Zurich, Schmelzbergstrasse 9, Zurich, 8092, Switzerland

**Keywords:** Pea protein, Pea protein concentrate, High-pressure rheology, Differential scanning calorimetry, Total dietary fiber content, Soluble dietary fibers, Insoluble dietary fibers, Sustainability, Protein functionality

## Abstract

Pea proteins are gaining increased interest from both the food industry as well as from consumers. Pea protein isolates (PPI) excel at forming meat-like textures upon heating while pea protein concentrates (PPC) are more challenging to transform into highly sought-after foods. PPCs are richer in dietary fibers (DF) and are more sustainable to produce than PPI. In this work, degradative enzymes were used to modify the functionality of PPC-water blends with a focus on texturization upon heating. Three enzyme solutions containing *β*-glucanases, hemicellulases, pectinases, xylanase, and cellulases were added to 65 wt% PPC blends. The effect of these enzymatic pretreatments was measured by monitoring the torque in a mixing reactor during blending, differential scanning calorimetry (DSC), high-pressure shear rheology (HPSR), and DF content and size analysis. Four endothermic peaks were detected in the DSC thermograms of PPC, namely at 63 °C, 77 °C, 105 °C and 123 °C. The first three peaks were attributed to phase transition and gelation temperatures of the starches and proteins constituting PPC. No endothermic peaks were measured for PPI blends. Enzyme solutions containing *β*-glucanases, hemicellulases, pectinases, and xylanases increased the endothermic energy of all peaks, hinting at an effect on the gelation properties of PPC. The same enzymes decreased the resistance to flow of PPC blends and induced a shift of the weight average molecular weight (Mw) distribution of soluble dietary fibers (SDF) towards smaller values while increasing the fraction of SDF by decreasing the insoluble dietary fiber (IDF) content. The solution containing cellulases did not change the DSC results or the viscosity of the PPC mixture, nor did it affect the IDF and SDF contents. On the other hand HPSR measurements of heated PPC samples up to 125 °C showed that all tested enzyme solutions decreased the complex viscosity of PPC-water blends to values similar to PPI-water blends. We demonstrated that degradative enzymes can enhance the functionality of less refined protein-rich ingredients based on pea and other vegetal sources. Using optimized enzyme blends for targeted applications can prove to be a key changer in the development and improvement of sustainable protein-rich foods.

## Highlights

• Pea protein concentrate functionalities were changed using degradative enzymes.
• Plant protein gelation investigated using high-pressure oscillatory shear rheology.
• Degrading dietary fibers of pea protein concentrate changes functionality.
• Hemicellulases, *β*-glucanases, pectinases or xylanases increase content of soluble dietary fibers.

## Graphical abstract

**Figure:**
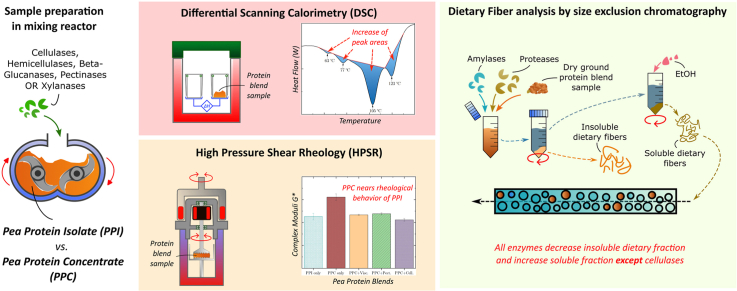

## 1 Introduction

Proteins are a crucial macronutrient for the human diet. They are needed to synthesize the proteins of the body that fulfill various vital roles such as for the function of vital organs and to build up muscles (Mittendorfer et al., 2020). The origin of these proteins has been progressively shifting from animal-based towards plant-based sources to sustain the growth of the world's population. Moreover, sustainable and animal welfare are driving the transition to alternative protein sources.

Soy and pea proteins are currently the most sought-after protein alternatives of botanical origin. Especially yellow pea (*Pisum sativum*) is recently gaining interest thanks to growing conditions suited for continental climates such as central Europe and northern America, and its relatively high protein content (Fuhrmeister and Meuser, 2003; Knott, 1987; Cousin, 1997). Dry pea protein powders can be classified into three groups that are determined by their protein fraction: Pea flour with 20 %–30 %, pea protein concentrate (PPC) with around 60 %–75 % and pea protein isolate (PPI) with 75 %–90 % proteins depending on environmental conditions, genetic factors and the method of fractionation (Millar et al., 2019; Daba and Morris, 2022; Kornet et al., 2020; Boye et al., 2010; Pelgrom et al., 2013).

Commercially available PPC is obtained by dry fractionation of dehulled and milled peas after they have been dried. The fractionation occurs by air classification making use of the weight difference of the larger starch granules as opposed to the smaller protein-rich fraction (Pelgrom et al., 2013). PPI, on the other hand, is produced by a wet separation process involving a solubilization step in alkaline conditions followed by a protein precipitation step around the isoelectric point of about pH 4.8 (Kornet et al., 2021). Consequently, proteins contained in PPC powders are less denatured than those of PPI due to the milder fractionation method. Moreover, the production of PPI requires large amounts of water in addition to bases and acids, making PPI less sustainable than PPC (Schutyser and van der Goot, 2011; Desiderio et al., 2023).

Among pea protein sources, PPI is the most used to produce protein-rich foods such as animal-free meat-like alternatives. Such meat analogs have gained increased interest as they have the potential to reduce the consumption of animal produce by mimicking the texture of these traditional products (Onwezen et al., 2021; Michel et al., 2021; Boukid, 2021). Such products are most often produced by cooking extrusion, involving the input of thermal energy and shear to generate desired proteinaceous fibrillar structures (Zink et al., 2023; Dekkers et al., 2018). Conversely, no successful texturization of PPC as the sole texturizing ingredient for meat-like structures can be found in the literature.

Indications on how to improve the applicability of pea protein fractions by increased gelation can be found in the literature. The presence of salt can improve the yield and viscosity of PPC by using milder extraction conditions (Kornet et al., 2020; Sun and Arntfield, 2010). Some studies observed that enzymatic treatments of pea proteins with transglutaminase were able to alter the rheological properties of pea protein mixtures towards a stronger, more elastic gel, similar to soy protein isolate (Shand et al., 2008; Sun and Arntfield, 2012).

In the scope of this work, the focus is laid on the improvement of the texturizing properties of PPC by enzymatic degradation of the fibers. Pea ingredients such as dietary fiber (DF) can affect the structure and texture of extruded snacks by, for example, altering their capacity to bind water or oil. This also depends on the type of fiber, where generally soluble DF, which is more likely to be found in the inner part of the pea, and insoluble DF, which is more likely to be found in the hull, can be distinguished (Tulbek et al., 2017). The polysaccharides of the endosperm and cotyledon of peas are, besides starch, cellulose and hemicelluloses, composed of ca. 55 % pectic polysaccharides, where arabinan and galacturonan are reported to be the most abundant glycans but also linear xylan, homogalacturonan, and rhamnogalacturonan have been detected (Brillouet and Carré, 1983; Patnode et al., 2019). Hemicelluloses that are present in the cell walls of all terrestrial plants include xyloglucans, xylans, mannans and glucomannans (Scheller and Ulvskov, 2010). Pectin is described as one of the most complex natural heteropolysaccharides since it can consist of up to 17 different monosaccharides and 20 different linkages. Homogalacturonan (HG) is generally recognized as the primary component and xylogalacturonan (XGA) and rhamnogalacturonan-I and –II (RG-I and RG-II) as further regions in the highly branched DF. However, the abundance of the various regions and therefore contents and molar ratios of the corresponding sugars can vary greatly depending on the origin and extraction method (Voragen et al., 2009; Noguchi et al., 2020). For example, Noguchi et al. (2020) observed that pectin extracted from a pea fiber preparation that branched arabinan, which was directly linked to the RG-1 region, accounted for ca. 50 % of the pectin content and the HG region only accounting for a small part.

PPC contain around 20 % of DF compared to only about 3 % in PPI (Shand et al., 2007). We hypothesize that these DFs have a considerable impact on the thermo-gelation properties of PPC. Consequently, changing the molecular weight of these DF could be key to improving the functionality of PPC.

We explored the impact three distinct degradative enzyme blends had on the rheological and thermal gelation properties of PPC in comparison to PPI. These blends contained cellulases, hemicellulases, pectinases, *β*-glucanases, and other enzymes that have the potential to degrade the DFs present in PPC. The relationship between rheological and thermophysical properties, and DF properties were investigated. For this, pea-based dough-like blends were mixed using a duplex kneader. By recording the torque needed for generating a homogeneous blend, it was possible to investigate the kinetic action of the enzymes on the flow properties of pea protein and water mixtures. The gelation properties of the so-produced pea protein blends were then analyzed using high-pressure shear rheology (HPSR) and dynamic scanning calorimetry (DSC). Finally, fiber analysis using size exclusion chromatography was used to draw conclusions between the fiber size distribution and the gelation properties of the proteinaceous blends. To our knowledge, it is the first time these measuring techniques have been used to investigate the rheological and thermophysical effect degradative enzymes have on pea protein sources. Moreover, the gelation and thermal properties of the pea protein blends were investigated under elevated static pressure conditions and temperatures up to 125 °C to approach authentic conditions appearing in high moisture extrusion cooking used for the manufacturing of pea protein-based meat analogs, which is higher than what can be found in the majority of publications that do not go above the evaporation temperature of water.

## 2 Materials and methods

### 2.1 Materials

Pea Protein Concentrate (PPC) powder (ca. 55 % pea protein and ca. 20 % pea fibers according to manufacturer) was obtained from Vestkorn Milling AS (Pea protein F55X, NOR). The comparative Pea Protein Isolate (PPI) powder (Nutralys F85G, 85 % pea protein and ca. 10 % pea fibers according to manufacturer) and the pea protein fibers (I50M, 1:1 pea fibres to starch) were sourced from Roquette Frères Lestrem (FR). Sodium azide was purchased from Fluka Chemie AG (CH). The three enzyme blends tested in this work, Viscozyme® L, Pectinex® Ultra SPL and Celluclast 1.5L® were obtained from Sigma-Aldrich GmbH (DK).

According to the manufacturer Novozyme and supplier Sigma-Aldrich, Viscozyme® L is an enzyme mixture containing a wide range of carbohydrolases, including arabanase, cellulase, *β*-glucanase, pectinases, hemicellulases, and xylanase with a declared activity in fungal *β*glucanase units (FBGU) of ≥100 FBGU/g and a density of 1.10–1.30 g/ml. Pectinex® Ultra SPL is a pectinase solution produced from *Aspergillus aculeatus* composed of mainly pectinases such as pectintranseliminase, polygalacturonase, and pectinesterase, and small amounts of hemicellulases, *β*-glucanases and cellulase with a declared activity of ≥3800 polygalacturonic acid units/mL. Celluclast 1.5L® is a cellulase blend from *Trichoderma reesei* and catalyzes the breakdown of cellulose into glucose, cellobiose, and higher glucose polymers with a declared activity in endoglucanase units of ≥700 EGU/g and a density of 1.10–1.30 g/ml.

In the scope of this work, we use the better readable terms of “Viscozyme”, “Pectinex” and “Celluclast” to refer to Viscozyme® L, Pectinex® Ultra SPL and Celluclast 1.5L®, respectively.

For the fiber analysis, the Total Dietary Fiber Assay Kit was ordered from Megazyme (IRL). Absolute ethanol, acetone, MES ≥99 %, Trizma Base ≥99.9 %, sodium azide ≥99.5 %, sodium hydroxide ≥98.0 % and sodium nitrate ≥99.0 % were purchased from Sigma-Aldrich Chemie. Ultrapure (Milli-Q) water was used for all analytical analyses (Merck Millipore, Merck KGaA, GER). The standards for the size exclusion chromatography, PolyCAL^TM^ polyethylene oxide (PEO-24 K, Mw = 23'651 g/mol, Mn = 23'432 g/mol, dn/dc = 0.132 mL/g, IV = 0.41 dL/g) and PolyCALTM dextran (DEX-70 K; Mw = 70'026 g/mol, Mn = 55'411 g/mol, dn/dc = 0.147 mL/g, IV = 0.260 dL/g) were purchased from Malvern Instruments (Malvern Panalytical Ltd, UK).

### 2.2 Sample preparation and mixing torque measurement

**Fig. 1:**
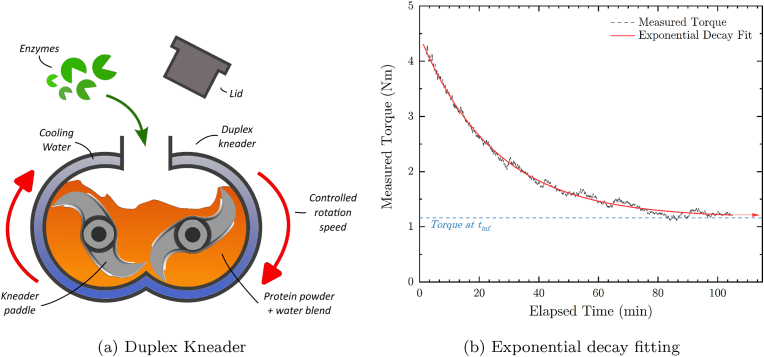
(a) Schematic representation of duplex kneader with kneading paddles, protein blend (orange), enzyme solutions (green) and cooling water (blue). The red arrows indicate the direction of the blade rotation. The lid used to minimize water evaporation during the measurement is shown on top of the representation in grey. (b) Selected example of simple exponential decay fit. Datapoints are shown in black dots, and the exponential fitting curve by a red line. The red arrow is a guide to the eye indicating the hypothetical final torque *τ*_*∞*_ at *t* = *∞* shown by a dashed blue line. (For interpretation of the references to color in this figure legend, the reader is referred to the Web version of this article.)

Pea protein-based dough-like samples were prepared using a lab-scale duplex kneader (MKD 0.6 H60, IKA Werke GmbH, DE) as depicted in Fig. 1a, favoring homogeneous mixing of the protein powder with water and enzyme solutions while allowing for torque monitoring over time. The rotational speed of the two intermeshing kneader blades was set to 50 rpm and the mixing time to 105 min for all formulations. The double jacket of the mixing chamber was temperature controlled by a water bath set to 25 °C to avoid excessive heat-up of the PPC blends due to viscous friction. The PPC samples with a dry mass fraction of 50 wt% to 65 wt% and water was produced by adding 0 wt% to 2 wt% of the three different enzyme mixtures. Samples made with 65 wt% PPI including 0.25 % viscozyme were also produced. Additionally, samples with PPI, pea fibers, and pea starch were blended to reconstitute PPC from its dry fractions. For this, 66 % of PPI were blended with 34 wt% of I50M for a PPI + Fiber blend with approximately 56 wt% pea proteins and 20 wt% pea fibers. The enzymatic solutions were mixed with the water fraction to ensure a homogeneous distribution within the viscous samples. Furthermore, the protein mixtures were prepared with 0.01 wt% sodium azide (Fluka Chemie AG, CH) to avoid microbial growth during the measurements. The so-produced dough-like samples were immediately stored at −20 °C in closed containers after their preparation to stop the enzymatic reactions before further analysis. All samples were produced and measured in triplicates.

The viscosity decay rate during mixing was recorded and fit with an empirical single-term decay function with a time constant to quantitatively compare the different formulations (Fig. 1b). This decay equation is shown by Eq. (1), whereby *τ*_*t*_ in *Nm* is the measured torque at an elapsed mixing time *t* in *s*, *τ*_*∞*_ is the hypothetical final torque representing the torque plateau after an infinite mixing time. The dimensionless normalization constant *A* allows for a relative comparison between different samples and *λ* stands for the exponential torque decay rate in *s*^−1^. Despite its relative simplicity, this fit yielded goodness of fits above 0.98 (R≥0.98) for all measurements.

(1) $$\tau_{t}=\tau_{\infty }+A\cdot e^{-\lambda t}$$

### 2.3 Differential scanning calorimetry DSC

**Fig. 2:**
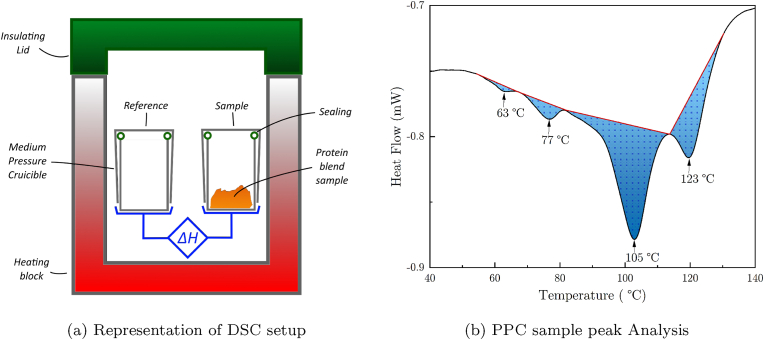
(a) Representative drawing of DSC setup. The samples were placed in a heating setup closed by a thermally insulating lid shown at the top in green. Two medium-pressure resistant crucibles with sealings were used to reduce water evaporation during the measurement. An empty crucible (left) was used as a reference while ca. 10 mg frozen dough-like protein samples were placed into the sample crucible (right). (b) Example of a DSC curve analysis. The surfaces of the four negative peaks shown in blue were interpolated between the measurement data (black) and visually determined boundaries (red lines) at the onsets and endsets of their respective peak. Black arrows are guide-to-the-eye to highlight the observed peak temperatures at 63 °C, 77 °C, 105 °C and 123 °C. The PPI DSC curves were devoid of such peaks. (For interpretation of the references to color in this figure legend, the reader is referred to the Web version of this article.)

The effect of the enzymatic treatment of PPC on the enthalpy change during heating was measured using a DSC 3+ device (Mettler Toledo AG, CH). For this, around 10 mg of frozen PPC dough-like samples were weighted and sealed into medium pressure-resistant crucibles to minimize water evaporation before and during the DSC measurement. The samples were immediately analyzed after weighing to minimize enzymatically induced changes. This is schematically depicted in Fig. 2a, also showing the empty sealed crucible that was used as a reference sample. The heating rate was set to 2.5 K/min and the measured temperature ranged from 25 °C to 140 °C. Higher temperatures only showed carbonization of the sample and were thus, not relevant for this work. Moreover, DSC measurements of a cooling step after the heat treatment did not yield additional information. Unlike PPI, which is known to show a virtually smooth DSC curve, the PPC samples exhibited four distinct peaks as shown and discussed in the results part in Fig. 6.

No clearly defined baseline could be distinguished for the PPC samples. Therefore, the enthalpy difference Δ*H* change was calculated by integrating the area under each peak delimited by a linear baseline between onset and endset selected visually for each of the four negative enthalpy peaks. These peaks were observed at around 63 °C, 77 °C, 105 °C and 123 °C for all PPC samples. An example of such an analysis is shown in Fig. 2. The surface under the peaks was analyzed using Origin 2021b (OriginLab Corporation, USA). The temperatures of the negative peaks were not influenced by the addition of enzymatic solutions.

### 2.4 High pressure oscillatory shear rheology

**Fig. 3:**
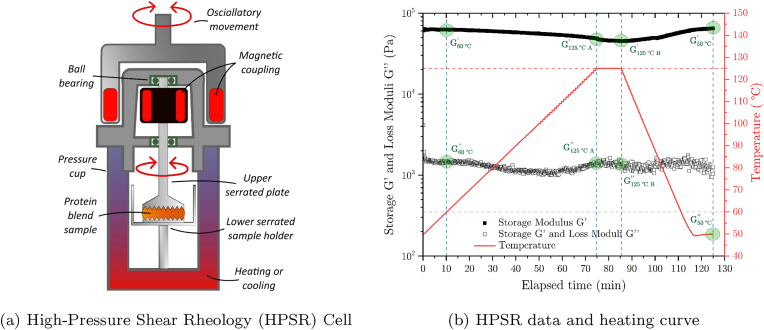
(a) Depiction of High-Pressure Shear rheology (HPSR) cell. The protein sample (orange) was analyzed using a closed serrated plate-plate geometry setup. Oscillatory movements of the rheometer were transmitted through a magnetic coupling (red) while the samples were heated and pressurized with N_2_ at 10 barg. (b) Samples were heated at a rate of 1 K/min and cooled at 2.5 K/min as shown by the red curve. An example of an HPSR measurement is shown by the full and empty black squares representing the storage (*G*′) and loss (*G*″) moduli, respectively. Green circles highlight extracted data points at the onset of the measurement at 60 °C, the beginning and end of the holding ramp at 125 °C, and at 50 °C after the cooling phase. (For interpretation of the references to color in this figure legend, the reader is referred to the Web version of this article.)

The viscoelastic properties of enzymatically treated PPC samples subjected to high temperatures were evaluated by High-Pressure Shear Rheology (HPSR) using a magnetically coupled high-pressure shear cell (MCR 302/CC25, Anton Paar GmbH, AT) equipped with a serrated plate-plate configuration (PP20/PR/SS/P2 and PP20/PR/P2, Anton Paar GmbH, AT) as schematically depicted in Fig. 3a. PPC and PPI samples were produced using a duplex kneader as previously described. The samples were prepared by deposing 1.8 g of the dough-like masses onto the center of the lower serrated plate, followed by a precise closing of the upper part of the setup so as not to smear out the sample. This procedure had to be followed precisely since the setup does not allow trimming the overage sample after lowering the upper geometry like during standard rheological procedures. The measurement of the storage (*G*′) and loss (*G*″) moduli of the proteinaceous samples subjected to a heating step were performed at an amplitude of 0.1 % and a frequency of 1 Hz. The heating step was performed as shown in Fig. 3b, whereby a heating ramp of 1 K/min was applied up to 125 °C followed by a holding time of 10 min before the samples were cooled down to 50 °C at a rate of 2.5 K/min with a final holding step of 10 min. These steps were performed using a Peltier element (C-PTD200, Anton Paar GmbH, AT) connected to a water bath (Julabo F10-VC-3, Julabo, DE). The denoted heating rate was selected to enable the sample to adapt to the heat flux, while the cooling rate was maximized to minimize sample changes after the holding step at 125 °C. To avoid sample water evaporation, nitrogen at 10 barg (99.995% purity, PanGas AG, CH) was injected into the high-pressure setup and upheld during the duration of the measurement.

Four distinct time-temperature points were extracted to capture the most relevant transition points: The dynamic moduli at (i) 60 °C at the onset of the heating ramp ($G_{60\;\circ \text{C}}^{\prime }$ and $G_{60\;\circ \text{C}}^{\prime \prime }$), (ii) at the beginning ($G_{125\;\circ \text{C}\;A}^{\prime }$ and $G_{125\;\circ \text{C}\;A}^{\prime \prime }$) and (iii) at the end ($G_{125\;\circ \text{C}\;B}^{\prime }$ and $G_{125\;\circ \text{C}\;B}^{\prime \prime }$) of the high-temperature heating ramp as well as iv) after the holding time following the cooling step ($G_{50\;\circ \text{C}}^{\prime }$ and $G_{50\;\circ \text{C}}^{\prime \prime }$). The highest temperature of 125 °C was chosen based on the results from the DSC analysis detailed in the previous section.

### 2.5 Fiber analysis

#### 2.5.1 Soluble and insoluble dietary fiber content

**Fig. 4:**
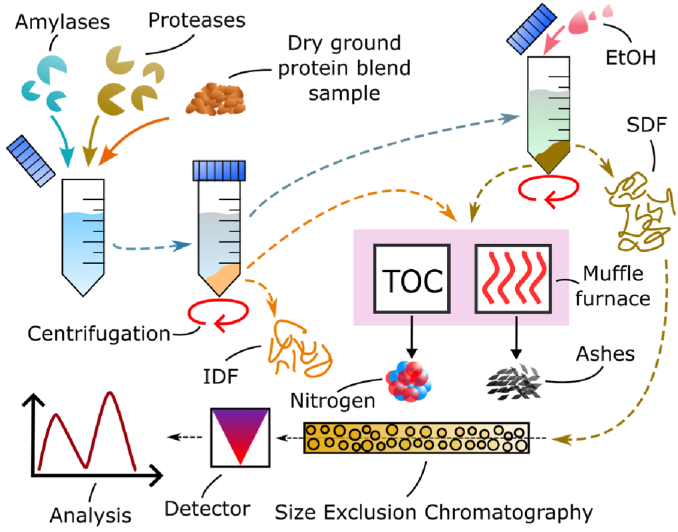
Enzymatic-gravimetric analysis of the total dietary fiber (DF) content. The pathways to extract insoluble (IDF) and soluble (SDF) DF are shown in orange and khaki, respectively. Ashes were measured using a muffle furnace. Nitrogen content was analyzed by TOC. The weight average molecular weight distribution of the SDF fraction was analyzed using size exclusion chromatography. (For interpretation of the references to color in this figure legend, the reader is referred to the Web version of this article.)

Triplicates of the frozen samples from the mixing torque measurement with 2 wt% enzyme concentration were freeze-dried (FreeZone 4.5 L −50 °C Benchtop Freezedryer, Labconco, USA) for 48 h and subsequently ground in a mortar. 1 g of each sample was weighed in duplicates. The analysis was performed according to the Total Dietary Fiber Assay description from Megazyme, except that the filtering steps were replaced by centrifugation. In this assay, starch and protein are digested enzymatically, the IDF residue is collected by centrifugation and the SDF residue by ethanol precipitation and subsequent centrifugation. The residues are quantified gravimetrically after drying and corrected for ash and protein contents. Ash content was determined by incineration of the fiber residues in a muffle furnace (Mod. L 51/S, Naber Industrieofenbau, CH) and protein content was calculated based on N x 6.25, where the total nitrogen content was measured using a TOC-L equipped with a TN module (Shimadzu Europe, GER) (see Fig. 4). Averages and standard deviations of the six obtained values per treatment type were calculated in Excel (Microsoft 365 Apps for Enterprise, Version 2311, Microsoft Corporation, USA) and plotted in Origin (2021) (OriginLab Corporation, USA).

#### 2.5.2 Molecular weight distribution of the SDF

The SDF residue of the total dietary fiber assay was further analyzed by size-exclusion chromatography (SEC) to estimate the weight average molecular weight distribution (Mw) of the SDF. A triple detection system (differential refractive index (RI), UV/Vis, light scattering (right-angle 90°(RALS) and low-angle (LALS, 7°)) detectors, as well as a viscometer) consisting of an OMNISEC resolve and an OMNISEC reveal module (Malvern Panalytical Ltd, UK) were used (see Fig. 4). The column system consisted of a pre-column (Viscotek AGuard Column, 0.50 cm × 6 mm) and two A6000M columns in series (Viscotek, 30 cm × 8 mm, Malvern Panalytical Ltd, UK), which were kept at 20 °C. The autosampler was kept at 60 °C. The flow rate was set to 0.7 mL/min and the injection volume was 100 μL. For the analysis, 20 mg of each sample were suspended in 10 mL SEC eluent (0.1 M sodium nitrate and 0.02 % sodium azide) to a concentration of 0.2 %w/v. Each sample triplicate was injected twice, therefore the solutions were kept stirring at 60 °C and 1.5 mL of the solution was filtered (0.45 μm, nylon) into an HPLC vial immediately before injection. Polyethylene oxide (PEO-24 K) and dextran (DEX-70 K) solutions were prepared in the SEC eluent as calibration and verification standards. To analyze the obtained data from the RALS/LALS, RI and viscosity detectors a dn/dc of 0.147 mL/g was used in the OMNISEC Software Version 10.30. For visualization, the RALS and RI signals of the six measurements per treatment type were averaged in Excel (Microsoft 365 Apps for Enterprise, Version 2311, Microsoft Corporation, USA) and plotted in Origin (2021) (OriginLab Corporation, USA).

### 2.6 Statistical analysis

Unless stated otherwise, all measurements were conducted in triplicates. The results of each data point were obtained by calculating the arithmetic mean and the standard deviation of each measurement point. These values were calculated using the spreadsheet editor software Microsoft Excel 2019; OriginLab 2020.

## 3 Results and discussion

### 3.1 Enzymatic action during blending

**Fig. 5:**
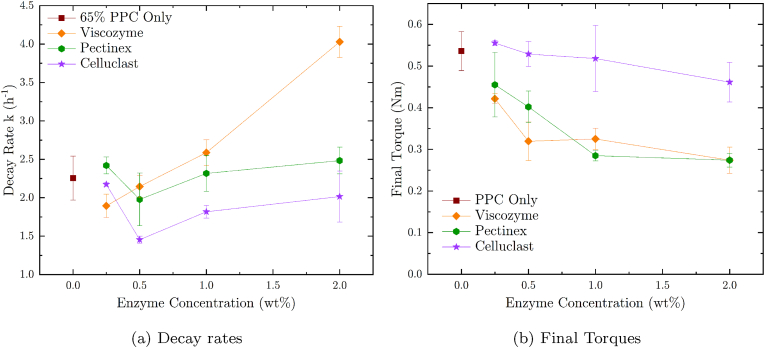
a) Decay rates *k* and b) final torques obtained from fitting single-term decay function to torque measurements of pea protein concentrate (PPC) blends. PPC blends were prepared with 35 % water and 0.25 %–2.0 % Viscozyme (orange diamonds), Pectinex (green circles), and Celluclast (purple stars) enzyme solutions. A control (brown squares) was also measured and fitted with the same equation. PPC blends were prepared with a duplex kneader equipped with a torque measurement sensor and a double jacket to keep blend temperatures at 30 °C during a mixing time of 105 min at 50 rpm. Microbial growth was inhibited by sodium azide addition. (For interpretation of the references to color in this figure legend, the reader is referred to the Web version of this article.)

The decrease in resistance to flow rate (*k* in *h*^−1^) and the torque at infinite blending time (final torque *τ*_*∞*_ in *Nm*) of the protein-water mixtures during mixing are shown in Fig. 5. These two parameters were obtained by fitting the measured torqued during blending with a normalized exponential decay function detailed in the method section (eq. (1)). This way, it is possible to monitor and verify when the enzymatic reaction stops impacting the resistance to flow. Moreover, it also enables to estimate the lowest attainable torque which is another way to indirectly measure how the addition of enzymes changes the viscosity of the protein-water blends. Similar approaches using viscosity measurement to follow enzymatic degradation were taken in other works (Tomlin et al., 2014; Tayal et al., 1997). Such change in viscosity is driven by three dominating mechanisms. At the beginning of the blending process, the dry components of the protein powders are (i) covered by the water phase forming bridges or a continuous phase between the particles. These water bridges can lead to strong attractive forces between the particles increasing the resistance to flow that is well documented in literature (Murase and Sekiguchi, 2019; Koos, 2014). In our case, the resistance to flow was mainly driven by particle-particle friction lubricated by the water phase. Thus, the viscosity of the protein-water slurry decreased upon mixing. (ii) It is well known that hydrophilic foods hydrate upon the addition of water leading to a swelling of protein, fibers, and starch particles but also to a breakage of larger aggregates and diffusion of molecules into the continuous water phase. This leads both to an increase in viscosity through water binding and to a decrease in resistance to flow by a reduction in Coulomb friction and lower deformation stresses of particles and aggregates. These effects have also been described and investigated in other works (Tulbek et al., 2017; Clavaud et al., 2017; Ji et al., 2022; Kew et al., 2021). Finally, (iii) the addition of fiber-degrading enzymes reduces the size of the DF or even leads to their breakdown into oligo- or monosaccharides and thus, acts towards an overall decrease of the viscosity of the pea protein-water blends (Ravn et al., 2015; Kumari et al., 2023).

**Fig. 6:**
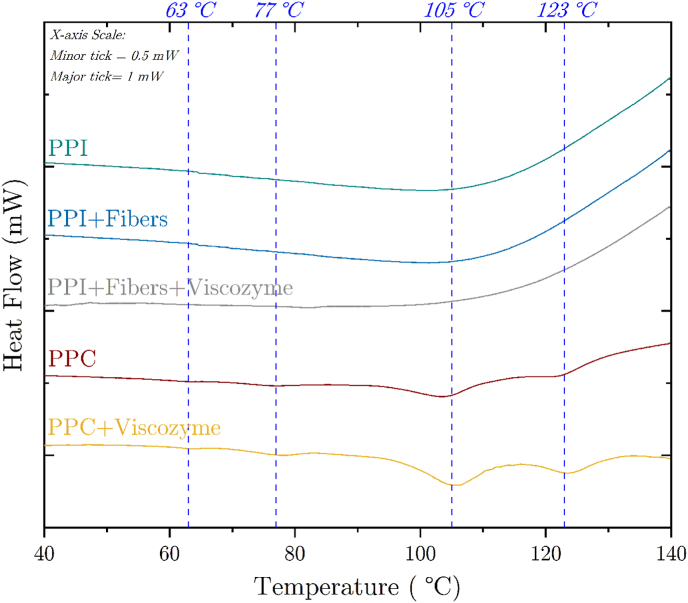
Thermograms of 65 wt% pea protein concentrate (PPC) and pea protein isolates (PPI) blends with or without the addition of Viscozyme. PPI samples (upper three curves) were produced with PPI only (turquoise line), and blended with pea fibers to match the composition of the PPC blend (cyan line). The later PPI mixture with fiber addition was also enzymatically treated with 0.25 % Viscozyme (grey line). The lower two curves depict PPC blends without (brown line) and with the addition of 0.25 % Viscozyme (mustard line). Vertical dashed lines (blue) are guide-to-the-eye for selected peak temperatures of 63 °C, 77 °C, 105 °C and 123 °C. The ordinate axis is at the same scale for all samples but curves were stacked for better visualization. The linear scale of the x-axis is 1 mW for the distance between two major ticks and 0.5 mW for minor to major ticks. The heating rate was set to 2.5 K/min. (For interpretation of the references to color in this figure legend, the reader is referred to the Web version of this article.)

The decay rate *k* and the final torque *τ*_*∞*_ of a pea protein concentrate (PPC) and water blend with a powder fraction of 65 % were measured to determine the effect of degradative enzymes acting on the DF contained in the protein powder and water blends. For this, a PPC blend without enzyme addition was measured along with PPC blends to which 0.25 wt% to 2.0 wt% of the three enzyme solutions, namely Viscozyme, Pectinex and Celluclast, were added. The Viscozyme enzyme solution showed the most pronounced impact on the decay rate *k* of the resistance to flow for the PPC blend as can be seen in Fig. 5a. The decay rate of Viscozyme-containing blends increased with higher addition levels up to the highest tested concentration of 2.0 %. The Pectinex blend did not change the decay rate whereas the Celluclast blend showed some tendency to decrease *k*. In other words, the decrease in the resistance to flow is accelerated by the Viscozyme blend while Pectinex and Celluclast did not have a relevant impact. On the other hand, the final torques of the PPC blends depicted in Fig. 5b, were equally lowered by both Viscozyme and Pectinex up to a concentration of 1.0 %, which seems to be near a concentration threshold for the highest possible viscosity decrease when using these enzyme blends in the given PPC mixture. This threshold is most probably determined by the time the enzymes are active under these conditions and their concentration in the blend along with their enzymatic activity. PPC mixture produced with Celluclast did not show such a clear decrease in blend viscosity and less a concentration threshold. In summary, it seems that the *β*-glucanases, pectinases, or hemicellulases contained within the Viscozyme and Pectinex blend have an impact on the viscosity of PPC blends. In contrast, the cellulases of the Celluclast solution only seem to have a negligible overall impact on *k* and viscosity reduction. The higher decay rates obtained with Viscozyme could be a result of the xylanases that are presumably not, or only in much lower concentrations, present in Pectinex. Here it has to be noted that it is highly challenging to conclude on any specific enzymatic activities as the PPC blends are too complex to consider the effect of all the components and the exact composition as well as specific enzyme activities of the three enzyme solutions are not declared by the manufacturer. It is therefore, not possible to attribute a specific effect to a particular enzyme or enzyme blend.

### 3.2 Differential scanning calorimetry

**Fig. 7:**
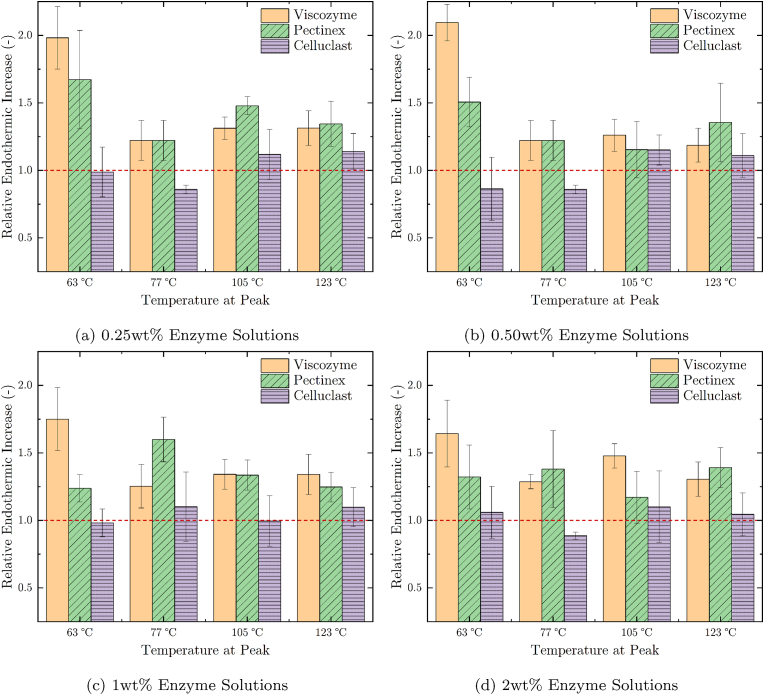
Relative endothermic peak increase of 65 % PPC samples with 0.25 wt% to 2 wt% Viscozyme, Pectinex or Celluclast when compared to an untreated PPC reference. The four peak temperatures of 63 °C, 77 °C, 105 °C and 123 °C were determined from DSC thermograms. Values ≃ 1 indicate no change compared to the reference. Relative endothermic values <1 or >1 indicate a decrease or increase, respectively. PPC samples prepared with Viscozyme are shown in mustard color, those blended with Pectinex in green, and the ones treated with Celluclast in purple. The red dashed line shows a relative endothermic increase value of 1. (For interpretation of the references to color in this figure legend, the reader is referred to the Web version of this article.)

The differential scanning calorimetry (DSC) results are separated into two parts. In the first part, DSC curves of pea protein concentrate (PPC) and pea protein isolate (PPI) blends are shown and discussed (Fig. 6). A more detailed analysis of the impact of the three enzyme solutions on the differential thermograms of PPC is discussed in the second part of this section and depicted in Fig. 7.

Differences between DSC curves obtained with PPI samples and PPC can be visually assessed by comparing the thermograms displayed in Fig. 6. PPI curves exhibited a smooth tendency without any exo- or endothermal peaks between 40 °C and 140 °C. This was to be expected as it is well known, that proteins contained within pea isolate powders have already undergone denaturation when isolated from the other pea constituents such as pea fibers and starch (Kornet et al., 2021; Sun and Arntfield, 2010). Similar smooth curves were also measured upon adding pea fibers and after enzymatic treatment with 0.25 wt% Viscozyme. On the other hand, DSC thermograms of PPC blends showed four distinct endothermal peaks at around 63 °C, 77 °C, 105 °C and 123 °C. Peak onset and conclusion temperatures were not taken into account due to a large uncertainty of the shape of the baseline. The peak temperatures shifted about 1 °C–2 °C towards higher peak temperatures when enzymatically treated with 0.25 wt% Viscozyme. The same was observed with the other enzyme solutions Celluclast and Pectinex but are not shown in this figure as they are very similar to the PPC + Viscozyme curve. Similar values were reported in literature with the first peak at around 63 °C being related to pea starch gelatinization or glass transition (Chung and Liu, 2012; Sim et al., 2019), and for the second peak at 77 °C which were related to pea protein denaturation (Shand et al., 2007; Sim et al., 2019; Mession et al., 2013) and temperature around 96 °C–110 °C related to the dissociation of amylose-lipid complexes (Eliasson, 1994) or the melting temperature of pea flour and water blends (Kristiawan et al., 2018). No literature data was found for the highest measured temperature peak at 123 °C. Given the complexity of the pea protein blends, we do not feel confident to further interpret these peaks. We will, therefore, refer to these endothermic peaks by mentioning their peak temperature rather than associating them with phase transitions.

The impact of the three tested enzyme solutions on PPC was evaluated by comparing enzymatically treated samples to the reference sample produced without any added enzymes. For this, the surface under each of the four peaks was integrated and divided by the surface of the reference sample as depicted in Fig. 7 for all four tested enzyme concentrations. Consequently, relative endothermic peak values ≃ 1 or <1 indicate that the enzymatic treatment did not change or even reduce the energy required to transform the PPC blend at the given peak temperature. Conversely, relative endothermic values >1 indicate that more energy was required to overcome the phase transition.

All four tested enzyme solution concentrations, i.e. 0.25 wt% depicted in Fig. 7a and 0.50 wt% shown in Fig. 7b and 1.0 wt% in Figs. 7c and 2.0 wt% in Fig. 7d showed the same tendencies. The Viscozyme and Pectinex solutions increased the endothermic energy of all identified peaks at 63 °C, 77 °C, 105 °C and 123 °C, whereas Celluclast had only a negligible impact. The concentration of all three enzyme solutions did not have an observable influence on this tendency. Furthermore, the highest increase in endothermic energy was measured at the first peak at 63 °C which is attributed to the gelation of pea starch.

### 3.3 High-pressure shear rheology (HPSR)

**Fig. 8:**
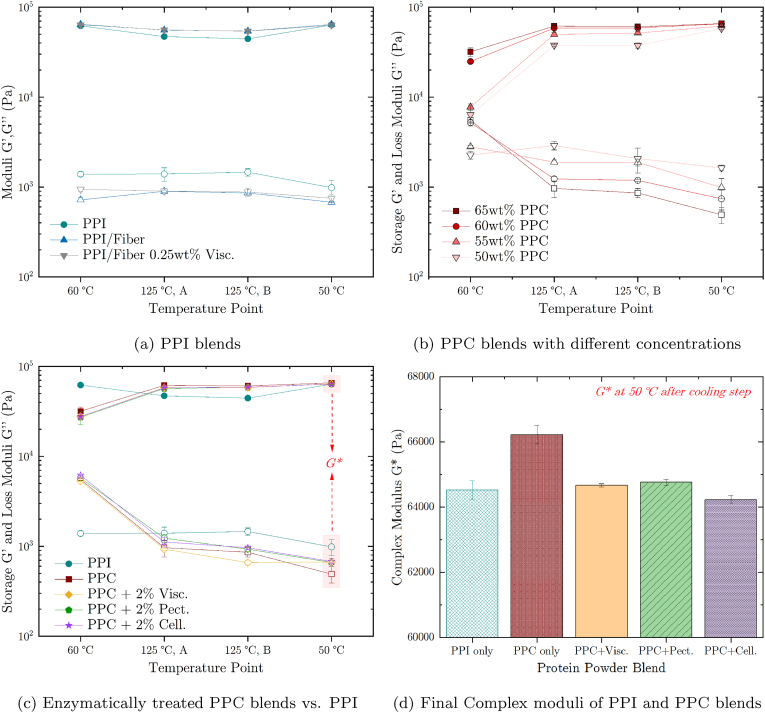
High-pressure shear rheology (HPSR) measurement results of pea protein isolate (PPI) and pea protein concentrate (PPC) blends. HPSR measurements were conducted while pea protein mixtures were heated up from 60 °C to 125 °C with a holding time of 10 min (125 °C, _*A*_ to 125 °C, _*B*_) before being cooled down to 50 °C. a) PPI-water blends with 65 wt% powder (turquoise circles) with added pea fibers (cyan pointing up triangles) and with 0.25 wt% Viscozyme on top (grey pointing down triangles). b) HPSR results of PPC mixtures with 50 wt% to 65 wt% powder (by increasing concentration: triangle down, triangle up, circle, square). c) 65 wt% PPI-water and PPC-water blends. Including Viscozyme, Pectinex and Celluclast solutions added at a concentration of 2 wt% to the PPC blends. *G*′ is depicted in full symbols, *G*″ in empty symbols in subfigures (a)–(c). d) Complex shear modulus (G*) calculated from the measurements shown in (c). (For interpretation of the references to color in this figure legend, the reader is referred to the Web version of this article.)

Rheological data on PPI and PPC blends with or without enzymes obtained using high-pressure shear rheology with a heating and cooling step is summarized in four plots in Fig. 10. The first of these plots (Fig. 8a) shows how the storage (*G*′) and loss (*G*″) moduli for 65 wt% PPI changes with the addition of fibers and with 0.25 wt% Viscozyme on top. The effect of the PPC concentration on the pea protein-water blends is depicted in Fig. 8b. The effect of the addition of 2 wt% Viscozyme, Pectinex and Celluclast to 65 wt% PPC blends is shown in Fig. 8c and d.

The change from a 65 wt% PPI only blend to a PPI mixture containing the same amount of powder but 20 wt% pea fibers decreased both *G*′ and *G*″. This was to be expected as the pea protein content was reduced from ca. 85 wt% down to 76 wt% due to the addition of the pea fibers. Therefore, the protein network strength formed during the heating step was weaker than the reference blend resulting in lower *G*′ and *G*″ values. Moreover, enzymatically treating this PPI blended with additional pea fibers did, apart from initially higher storage moduli, not significantly change the moduli compared to the untreated blend. Again, this result was expected as DSC analyses showed that adding 0.25 wt% Viscozyme seems not to change the physical properties of PPI even in the presence of additional pea fibers. Increasing the PPC fraction (Fig. 8b) resulted in higher *G*′ and *G*″ responses. This did not only confirm the well-known effect the increase of dry fractions has on the visco-elastic behavior of proteinaceous blends but is also proof that the HPSR method developed in the scope of this work can be used to analyze pea protein structure formation upon heating. Finally, Fig. 8c and d shows that the visco-elastic properties of PPC blends can be enzymatically treated to yield desired properties. Indeed, the complex modulus (G*) of PPC blends subjected to a heat treatment of up to 125 °C is higher than the G* of the target PPI blend. But by enzymatically treating the same PPC blend by adding 2 wt% of the Viscozyme, Pectinex, and Celluclast solution, the G* was overall reduced. Such an effect was expected for the Viscozyme and Pectinex enzymes but not from the cellulase of the Celluclast solution as the latter showed significantly less effect during torque and DSC measurements. Here, it would be interesting to investigate whether treating PPC with other non-degradative enzymes such as transglutaminase would increase G* as expected.

### 3.4 Fiber analysis

#### 3.4.1 Soluble and insoluble dietary fiber content

**Fig. 9:**
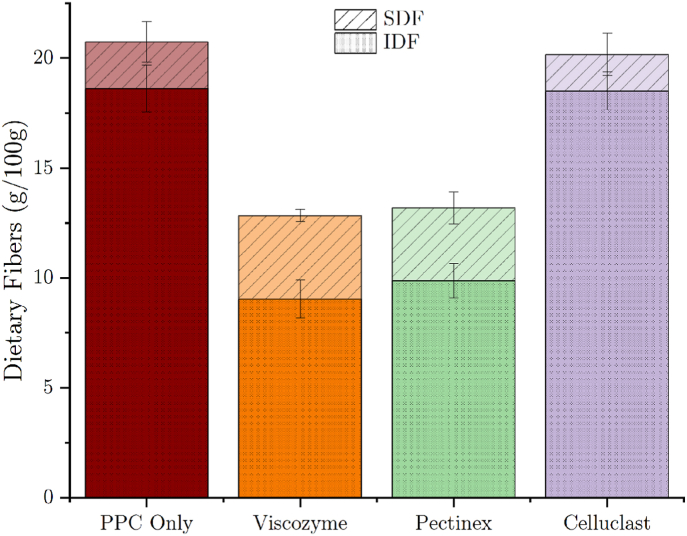
Content of soluble and insoluble dietary fiber per 100 g of freeze-dried PPC dough samples with or without enzyme treatment ± SD (n = 3).

The soluble (SDF) and insoluble dietary fiber (IDF) content was determined with the Total Dietary Fiber Assay Kit from Megazyme. The addition of 2 % Viscozyme and Pectinex enzyme solutions to the PPC blend led to a decrease by 51 % and 47 % in IDF compared to the blend where no enzyme solution was added (from 18.6 to 9.0 g/100 g and 9.9 g/100 g, respectively), while the content of SDF increased by 80 % and 57 %, respectively (from 2.1 to 3.8 g/100 g and 3.1 g/100g, respectively) (Fig. 9). It is assumed that primary cell walls are composed of interacting networks of xyloglucan/cellulose, pectin/cellulose, and proteins (Zykwinska et al., 2008), and also the pectic polysaccharides contained in pea cotyledon DF are suggested to be associated with cellulose (Brillouet and Carré, 1983). Therefore the degradation of one component could lead to a solubilization of another component. The reduction in the TDF (IDF + SDF) content could be caused by the enzymatic breakdown of the DF into mono-, di-, and oligosaccharides. Furthermore, it is important to take into account that in this assay, the collection of the SDF is performed by precipitation of the fibers with 80 % ethanol. Smaller SDF can remain soluble in the aqueous ethanol and, therefore, are not included in the final SDF residue. The addition of 2 % Celluclast enzyme solution to the PPC blend did decrease the content of SDF by 22 % (1.65 g/100 g) but did not affect the IDF content (reduction by 1 %–18.5 g/100 g). This observation suggests a low content of cellulose in the PPC blend which is in line with the manufacturers’ indication that the PPC blend contains IDF and SDF consisting mainly of pectic substances and hemicelluloses. Likewise, the PPC is produced from dehulled peas and cellulose is generally more abundant in the outer layers of peas. Furthermore, as opposed to the other two enzyme solutions, Celluclast contains only cellulases, limiting the range of activity on different types of DF. However, it is important to bear in mind the complexity of the constitution of plant cell wall polysaccharides and the activities of carbohydrate-degrading enzymes, including the not clearly declared composition and activities of the enzyme solutions applied in this work before drawing conclusions.

**Fig. 10:**
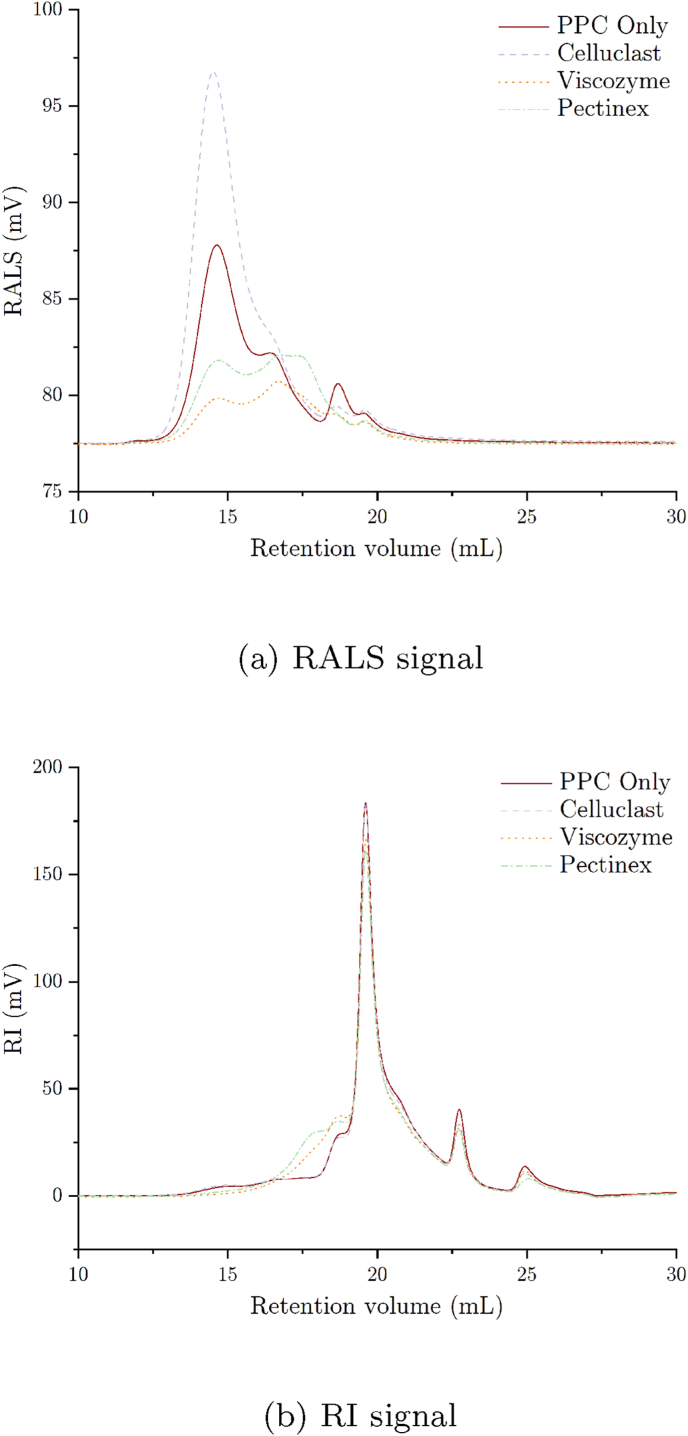
SEC right angle light scattering (RALS) and refractive index (RI) signal of the soluble dietary fiber fraction from the PPC dough samples with or without enzyme treatment (average n = 6) of sample triplicates injected in duplicates.

#### 3.4.2 Molecular weight of soluble dietary fibers

To estimate the size of the SDF in the samples, the SDF residue of the Total Dietary Fiber Assay was analyzed by SEC. Treating the PPC dough with Pectinex and even more so with Viscozyme enzyme solution tended to reduce the size of the SDF, which can be seen in the right angle light scattering (RALS) signal (Fig. 10a) in the shift of the curves towards a higher retention time. RALS detectors are primarily sensitive to the size of molecules. Attention should be drawn to the refractive index signal (Fig. 10b), which is more sensitive to the concentration of the respective sample fraction. It indicates that the biggest fraction of the SDF is present as smaller fibers, while the largest fibers make up only a small portion of the sample. To get a rough estimate of the Mw, an integration was performed over all peaks. On average, the enzymatic treatment with Pectinex and Viscozyme solutions resulted in a decrease of the Mw from 43 ± 4 kDa (untreated sample) to 30 ± 5 kDa and 21 ± 4 kDa, respectively. The apparent increase in the size of the DF from the Cellulase-treated PPC to 69 ± 21 kDa could be attributed to aggregation of the fibers in the sample, the release and solubilization of higher molecular weight fibers from the cell matrix, or a combination thereof. Aggregation of DF in aqueous solutions and the resulting overestimation of their size in SEC with light scattering detectors is for example known from the analysis of cereal *β*-glucan (Li et al., 2011). The TDF assay results showed no change in the IDF as well as SDF composition and the very high standard deviation of the Mw data could be an indicator of aggregation phenomena in this sample. But again, owing to the complexity of the polysaccharide compositions and interactions as well as the enzymatic mixtures the possible release of higher molecular weight dietary fibers can not be ruled out with certainty. In summary, Pectinex and Viscozyme had a similar impact on the DF parameters analyzed with the TDF assay and SEC, while the Celluclast solution did not seem to affect the DF to a relevant extent. Since the Pectinex as well as the Viscozyme enzyme blends contain similar enzymes, namely *β*-glucanases, hemicellulases, and pectinases it is reasonable that their impact on the DF is similar. According to the available information about the enzyme solutions compositions, Viscozyme additionally contains arabinases and xylanases that degrade arabinans and xylans. As previously described, it is assumed that arabinan is a major component in pea pectin, and xylan is a hemicellulose of plant cell walls. Therefore, the additional presence of arabinases and xylanases in Viscozyme could potentially explain the somewhat greater effect on the content of IDF and SDF as well as the size of the latter.

## 4 Conclusions

Addition of enzyme solutions containing *β*-glucanases, hemicellulases, pectinases, and xylanases decreased the viscosity of pea protein concentrate (PPC) blends. Moreover, these solutions increased the endothermic energy measured during DSC analysis of the PPC blends. The enzymatic treatment resulted in a reduction in insoluble dietary fiber (IDF) while increasing the soluble dietary fiber (SDF) fraction. Moreover, these enzyme solutions decreased the weight average molecular weight (Mw) of SDFs. On the other hand, the cellulase solution used in the scope of this work had only a negligible impact on the resistance to flow of PPC blends and endothermic peak energy. Consequently, no changes in the DF composition could be observed. Unexpectedly, high-pressure shear rheology (HPSR) measurements during heating followed by cooling of enzymatically treated PPC blends revealed that despite the previously described results, all used enzyme solutions decreased the complex viscosity of PPC blends to levels similar to the reference pea protein isolate blend. All in all, this work showcased the use of HPSR to measure the gelation properties of protein-rich foods, and described the impact of different fiber-degrading enzymes on PPC, while giving ideas on how to improve the functionality of less refined plant protein products. The latter products are more challenging to valorize but might prove to be an excellent source of dietary fiber while also being more sustainable than protein isolates.

## Author contributions

All authors contributed substantially to this research. JZ, OZW, LN and EW developed the conceptual framework. JZ and OZW developed the scenarios and methodological framework. JZ and DD measured and analyzed the HPSR, DSC and blender data. OZW measured and analyzed the Fiber data. JZ, OZW, LN and EW interpreted the data. JZ and OZW wrote the initial manuscript. DD, LN and EW edited and commented on the manuscript. LN and EW supervised the project.

## Declaration of competing interest

The authors declare that they have no known competing financial interests or personal relationships that could have appeared to influence the work reported in this paper.

## Data Availability

Data will be made available on request.
